# Comparison of morbidity and mortality of abdominoperineal resection vs low anterior resection in rectal cancer

**DOI:** 10.25122/jml-2026-0001

**Published:** 2026-03

**Authors:** Maria-Manuela Răvaș, Hortensia-Alina Moisa, Eugen Brătucu, Virgiliu Prunoiu, Mircea-Nicolae Brătucu

**Affiliations:** 1 Clinical Department No. 10, General Surgery, Carol Davila University of Medicine and Pharmacy, Bucharest, Romania; 2 Department of Oncological Surgery, Prof. Dr. Al. Trestioreanu Oncological Institute, Bucharest, Romania; 3 Department of General Surgery, Carol Davila Nephrology Clinical Hospital, Bucharest, Romania

**Keywords:** anterior rectal resection, abscess and fistula, rectal neoplasm, peritonitis, sepsis, RC, rectal cancer, LAR, low anterior resection, APR, abdominoperineal resection, AR, anterior resection, IR, inferior rectum, MR, middle rectum, SR, superior rectum, JR, rectosigmoidian junction, DM, diabetes mellitus, HTN, hypertension, TME, total mesorectal excision, LRRC, locally recurrent rectal cancer, BMI, body mass index, ICG, indocyanine green fluorescence

## Abstract

Postoperative sepsis is a serious complication of rectal cancer surgery and contributes to increased morbidity and mortality. This study aimed to identify risk factors and etiologies associated with postoperative sepsis in patients undergoing rectal surgery. A retrospective cohort study was conducted at a single center, including patients with rectal cancer who underwent surgery between November 2018 and February 2023. Tumors located <5 cm from the anal verge were treated with abdominoperineal resection (APR). Recorded variables included age, sex, surgical approach, tumor location, comorbidities (cardiovascular disease, diabetes, obesity), and loco-regional septic complications (fistula, abscess). A *P* value < 0.05 was considered statistically significant. A total of 226 patients underwent APR or low anterior resection (LAR). APR was associated with higher odds of early (OR = 1.84, 95% CI, 0.52–6.53; *P* = 0.14), late (OR = 2.75, 95% CI, 0.81–9.39; *P* = 0.13), and overall septic complications (OR = 2.38, 95% CI, 0.96–5.91; *P* = 0.06) compared with LAR; however, these differences were not statistically significant. After LAR, anastomotic leakage was the leading cause of postoperative sepsis (4.67%), including five early (<7 days) and three late (>7 days) fistulas. In the APR group, two patients developed late pelvic abscesses. Parastomal hernia was the most common late complication after APR. Postoperative loco-regional sepsis was more frequent in patients older than 50 years and in those with comorbidities, although no statistically significant association was observed overall. The median hospital stay was 11.7 days.

## INTRODUCTION

Rectal cancer (RC) is a form of colorectal cancer that affects approximately 1.9 million people annually worldwide, accounting for nearly 930,000 deaths globally [1]. The American Cancer Society reported an estimated 27,950 new rectal cancer cases in men and 19,000 in women in 2025, for a total of 46,950 new cases. From 2012 to 2021, incidence rates increased by 2.4% annually among younger individuals (<50 years of age) and decreased by approximately 1% among older individuals, largely due to the implementation of screening programs, particularly among individuals aged 50 years or older [2]. Mortality data for colon and rectal cancer are often combined because many rectal cancer–related deaths are misclassified as colon cancer. In the United States, an estimated 52,900 deaths from colorectal cancer are expected in 2025 [3]. In the European Union, colorectal cancer is the second leading cause of cancer-related death among males, with a mortality rate of 14.3 per 100,000, while among females it follows lung cancer as a leading cause of cancer mortality [4].

The rectum derives its name from the Latin term *intestinum rectum*, meaning “straight intestine.” It is an important distal portion of the large intestine and plays a key role in the temporary storage of feces, the regulation of defecation, and the maintenance of continence. Anatomically, the rectum begins at the level of the third sacral vertebra (S3), near the sacral promontory, as a continuation of the sigmoid colon. It measures approximately 12–15 cm in length and is divided into three segments: the superior, middle, and lower rectum, based on their distance from the anal verge [5]. The peritoneum covers the upper third of the rectum anteriorly and laterally, the middle third only anteriorly, and is reflected at the level of the lower third, forming the rectouterine pouch in women and the rectovesical pouch in men. Consequently, the lower rectum is extraperitoneal and covered by the fascia propria, which is the region where anastomosis is performed after low anterior resection (LAR). Posteriorly, the rectum is attached to the presacral fascia by Waldeyer’s fascia, beginning at the level of S4 [6].

Total mesorectal excision (TME) with rectal preservation is considered the gold standard treatment for rectal cancer [7]. When combined with negative histopathological margins, TME leads to favorable functional outcomes, improved healing, and lower local recurrence rates. Anterior resection with mesorectal excision has become the preferred surgical treatment for rectal cancer, as TME is associated with reduced local recurrence and improved survival.

Low anterior resection (LAR) and abdominoperineal resection (APR) are among the main surgical procedures performed for rectal cancer. The choice of surgical technique depends on patient-related factors, tumor location, and sphincter involvement. While LAR and AR preserve sphincter function and intestinal continuity, they carry a significant risk of anastomotic leakage. In contrast, APR eliminates the risk of anastomotic failure but is associated with specific complications related to the stoma (parastomal hernia, prolapse, necrosis) and perineal wound healing [7,8].

Although both procedures are associated with significant septic morbidity, the patterns, timing, and severity of sepsis differ between LAR and APR. Anastomotic leakage after LAR has been shown to significantly increase postoperative mortality and negatively affect oncologic outcomes, including local recurrence [9]. Conversely, septic complications after APR tend to be more localized but may result in prolonged morbidity and delayed recovery. Patient-related factors such as advanced age, male sex, diabetes mellitus, hypertension, and other comorbidities further increase the risk of postoperative sepsis in both surgical approaches [10,11].

Despite the clinical importance of postoperative sepsis, comparative data evaluating septic morbidity and mortality between LAR and APR remain limited and inconsistent. A better understanding of procedure-specific risks and contributing factors is essential to improve surgical decision-making, perioperative management, and patient outcomes.

Sepsis is a severe clinical syndrome associated with high morbidity, mortality, and substantial healthcare costs, estimated at approximately 45 billion US dollars annually in the United States for hospital-acquired infections alone [12]. The development of sepsis secondary to anastomotic fistula following colorectal surgery can be catastrophic, potentially progressing to generalized sepsis and death. Postoperative sepsis represents a major surgical complication and a significant healthcare burden. According to the literature, more than 50% of postoperative sepsis cases occur after gastrointestinal, thoracic, and cardiovascular procedures [13]. Patients who develop postoperative sepsis have an estimated mortality rate of approximately 25%. Identified risk factors include male sex, race, advanced age, and comorbidities such as diabetes mellitus, chronic renal disease, chronic liver disease, and hypertension [14].

Although the overall mortality associated with fistulas has decreased due to improved management, patients continue to die from fistula-related complications. Pelvic and intra-abdominal sepsis remains a leading cause of death, with a reported mortality rate of 0.5–1% [6]. Several studies have shown that anastomotic fistulas increase local recurrence rates after curative surgery, resulting in a significant negative impact on quality of life and life expectancy. Locally recurrent rectal cancer (LRRC) occurs in approximately 6–12% of cases, independent of chemotherapy [15]. Recurrence rates are highest in tumors of the lower rectum, followed by the middle rectum, and lowest in the superior rectum (50.9%, 30%, and 17%, respectively) [16].

Multiple studies have demonstrated that anastomotic leakage is strongly associated with patient comorbidities and surgical technique [13-15]. In particular, assessment of bowel perfusion using indocyanine green fluorescence has shown benefit, especially in patients with vascular disease or a history of embolic events that may impair local vascularization [17]. This technique reduces the risk of local sepsis by enabling early identification of ischemic bowel segments and preventing future anastomotic leakage. Tumor-related factors, such as stenosis, have also been identified as risk factors for anastomotic leakage and are associated with higher recurrence rates [18]. Early findings by Heald *et al*. demonstrated that most rectal carcinomas in the lower third could be treated with sphincter-conserving surgery [19].

Therefore, the aim of this study was to retrospectively compare septic morbidity and mortality following low anterior resection and abdominoperineal resection for rectal cancer and to identify clinical, surgical, and pathological factors associated with the development of postoperative pelvic sepsis.

## Material and Methods

The study was designed as a retrospective, single-center cohort including 226 patients diagnosed with rectal cancer and treated at the Department of Surgery of the Bucharest Oncological Institute “Prof. Dr. Alexandru Trestioreanu” over 5 years, from February 2018 to February 2023. The study included patients who underwent LAR with restoration of bowel continuity and patients who underwent APR with definitive stoma formation.

During the study period, surgeries were performed by the same surgical team using open or laparoscopic approaches, with most patients undergoing open surgery through a midline laparotomy incision. Rectal mobilization was performed with careful dissection to preserve the visceral pelvic fascia. TME was performed in most patients with mid- and low-rectal tumors.

### Surgical technique

Preoperative bowel preparation was carried out using oral Fortrans solution on the day before surgery. Patients were placed in the Lloyd–Davies position, and a urethral catheter was inserted after induction of anesthesia. Rectal dissection was performed under direct visualization. The mesorectal fascia and mesorectum were preserved intact, and the pelvic autonomic nerve plexuses were identified and preserved whenever possible. The lateral ligaments were divided bilaterally, followed by incision of the peritoneum approximately 2 cm above the rectouterine or rectovesical pouch. Below the Douglas pouch, Denonvilliers’ fascia was incised in male patients to separate the rectum from anterior structures, while in female patients, the rectum was separated from the posterior vaginal wall. After mobilization, rectal excision was performed at least 2 cm distal to the tumor using a transverse stapler. Mechanical transanal coloanal anastomosis or hand-sewn anastomosis was then constructed. A circular stapler was introduced transanally to perform a double-stapling anastomosis. Protective measures were applied selectively, particularly in patients with tumors located below the Douglas pouch. A diverting stoma, most commonly an ileostomy, was used as the primary protective method; transverse colostomy or transanal tube placement was also used in selected cases with favorable outcomes. In patients with tumors of the inferior rectum located less than 5 cm from the anal verge, APR was performed. This two-stage procedure consisted of an abdominal phase followed by a perineal phase, involving removal of the distal rectum, anal canal, anus, and surrounding sphincter muscles, with formation of a permanent colostomy, in accordance with abdominoperineal resection.

### Outcomes

The study aimed to evaluate the incidence of local pelvic septic complications (fistula and abscess) in patients undergoing low anterior resection/anterior resection or abdominoperineal resection for rectal cancer. Outcomes were assessed within 30 days postoperatively. The primary endpoint was the occurrence of anastomotic leakage within 30 days after LAR and the occurrence of pelvic abscess within 30 days after APR. Secondary endpoints included comparison of overall postoperative complications between the LAR and APR groups, such as ventral hernia, parastomal hernia, perineal hernia, mechanical ileus, and sexual dysfunction, occurring within 30 days after surgery. Complications were classified as early or tardive. Complications occurring within 7 days after surgery were defined as early, while those occurring more than 7 days postoperatively were classified as late complications.

### Statistical analysis

Fisher’s exact test was used to compare categorical variables between subgroups. A *P* value of less than 0.05 was considered statistically significant. The Mann–Whitney U test was used to compare continuous variables between groups. All statistical analyses were performed using SPSS software (Statistical Package for the Social Sciences), version 26.0 (IBM Corp., Armonk, NY, USA).

## Results

A total of 226 patients who underwent surgery for rectal cancer were included in the study; 55 patients (24.3%) underwent APR, and 171 patients (75.7%) underwent LAR. Among the LAR group, 61 patients underwent a Hartmann’s procedure due to incomplete LAR, followed by a second-stage restoration of bowel continuity. Additionally, anterior resections above the Douglas pouch were included.

The mean age of the study population was 63.1 ± 11.5 years, with no significant difference between the APR and LAR group (61.5 ± 10.0 vs 63.4 ± 12.2 years, *P* = 0.76). Male patients were predominant in both groups, accounting for 66.3% of the total cohort (69.1% in the APR vs 65.6% in the LAR, *P* = 0.76). No significant differences were observed regarding rural or urban residence between the two groups.

Comorbidities, including hypertension, diabetes mellitus, and obesity, were common and may have influenced postoperative outcomes. Obesity did not differ significantly between the APR and LAR group (16.4% vs 11.6%, *P* = 0.43). In contrast, diabetes mellitus (DM) was significantly more frequent in patients who underwent APR compared with those undergoing LAR (30% vs 9.9%, *P* = 0.0004). Cardiac disease (HTN) was similar between the two groups (67.3% in APR vs 58.8% in LAR/AR, *P* = 0.18).

The mean length of hospital stay for the entire cohort was 11.75 ± 7.55 days, with no statistically significant difference between the APR and LAR groups (11.35 ± 8.03 vs 13.5 ± 5.5 days, *P* = 0.08). Anastomotic leakage occurred exclusively in the LAR group, with an incidence of 4.67%. Overall postoperative mortality in the LAR group was 1.4% (three deaths), whereas none occurred in the APR group; however, this difference was not statistically significant (*P* = 0.55).

Neoadjuvant chemotherapy was administered to 75.1% of patients overall and was significantly more common in the APR group compared with the LAR group (88% vs 71.3%, *P* = 0.017). Similarly, neoadjuvant radiotherapy was more frequently used in patients undergoing APR (92% vs 74.9%, *P* = 0.009; Table 1). Within the APR group, 42% of patients (*n* = 21) received a radiotherapy dose of 50.4 Gy, 18% (*n* = 9) received 45 Gy, and 10% (*n* = 5) received 50 Gy. Evidence of tumor regression following neoadjuvant therapy was documented in 56% (*n* = 28) of cases. Tumor invasion of the anal canal was present in 42% of patients (*n* = 21), reflecting the locally advanced nature of disease in this subgroup (Table 2).

**Table 1:** Clinicopathological characteristics

Variable	All (*n* = 226)	APR (*n* = 55)	LAR (*n* = 171)	*P* value
Age, years, mean ± SD	63.1 ± 11.5	61.53 ± 10.05	63.4 ± 12.2	0.76
Sex, *n* (%)				
Men	150 (66.3%)	38 (69.09%)	112(65.6%)	0.76
Women	76 (34.3%)	17(34.0%)	59(34.5%)	
Comorbidities, *n* (%)				
Obesity	29 (12.8%)	9 (16.4%)	20 (11.6%)	0.43
Diabetes	32 (14.5%)	15(30%)	17 (9.9%)	0.0004
Cardiac history	137 (60.6%)	37(67.27%)	100 (58.8%)	0.18
Pathological stage post-chemoradiotherapy, *n* (%)				
Stage 0/I	48 (21.20%)	22 (40%)	26 (15.2%)	
Stage II	52 (29.40%)	11 (22%)	41 (23.96%)	0.0005
Stage III	101 (44.60%)	18 (32.7%)	83 (48.54%)	
Stage IV	25 (11.06%)	4 (7.3%)	21 (12.28%)	
Tumor distance from the anal verge				
Inferior rectum (0-6 cm)	73 (33.62%)	55 (100%)	18 (7.9 %)	
Middle rectum	95 (42.03%)	0	95 (42.03%)	0.001
Superior rectum/rectosigmoid junction	58 (25.66%)	0	58 (25.66%)	
Neoadjuvant therapy, chemotherapy *n* (%)	166 (75.1%)	44 (88%)	122 (71.3%)	0.017
Neoadjuvant radiotherapy, *n* (%)	174 (78.7%)	46 (92%)	128 (74.9%)	0.009
Hospital days	11.75 ± 7.55	11.35 ± 8.03	13.5 ± 5.5	0.08
Anastomotic leakage			4.6%	
Postoperative mortality, *n* (%)	3 (1.4%)	0	3	0.55

**Table 2:** Clinical, pathological, and laboratory characteristics of patients undergoing APR

Variable	Category	Value, n (%,)
Neoplasm type	ADK	50 (100%)
Tumor localisation	I.R	50 (100%)
Cancer invasion (tumor invasion of the anal canal)	Present	21 (42%)
Protective colostomy (before radiotherapy)	Yes	33 (66%)
Total circumferential rectal wall thickening	Yes	35 (70%)
Radiotherapy dose (45 GY)	Yes	9 (18%)
Radiotherapy dose (50 GY)	Yes	5 (10%)
Radiotherapy dose (50.4 GY)	Yes	21 (42%)
Tumor regression	Yes	28 (56%)

### Early postoperative complications occurring within less than 7 days

Early general postoperative complications occurred in four patients (7.2%) in the APR group and in 22 patients (12.9%) in the LAR group, with no statistically significant difference between groups (OR = 0.53, 95% CI, 0.17–1.61; *P* ≈ 0.34). Early septic complications were observed in four patients (8.0%) following APR and in seven patients (4.1%) following LAR/AR. This difference did not reach statistical significance (OR = 1.84, 95% CI, 0.52–6.53; *P* ≈ 0.14).

### Tardive postoperative complications occurring 7-30 days

Tardive general complications were reported more frequently in patients undergoing LAR (16.4%) than in those undergoing APR (14.5%), although this difference was not statistically significant (OR = 0.87, 95% CI, 0.37–2.04; *P* ≈ 0.9). Tardive septic complications occurred more frequently in the APR group (5 patients, 9.1%) compared with the LAR group (six patients, 3.5%), although this difference was not statistically significant (OR = 2.75, 95% CI, 0.81–9.39; *P* ≈ 0.13).

### Total septic complications and total complications

Considering both early (<7 days) and tardive (>7 days) events, a total of 13 patients (7.62%) developed septic complications. APR was associated with a higher rate of total septic complications than LAR (OR = 2.38, 95% CI, 0.96–5.91; *P* ≈ 0.06), indicating borderline significance. Total postoperative complications were also more frequent in the APR group, occurring in 30 patients (54.5%) compared with 63 patients (36.8%) in the LAR group (OR = 2.06, 95% CI, 1.12–3.81; *P* ≈ 0.06; Table 3).

**Table 3:** Postoperative complications: APR vs. LAR

GROUP	APR, *n* = 55	LAR, *n* = 171	*P* value
Early general surgical complication	4 (7.2 %)	22 (12,9%)	0.34
Early septic surgical complications	4 ( 8%)	7 (4.1%)	0.14
Tardive general surgical complications	8 (14.5%)	28 (16.4%)	0.9
Tardive septic surgical complications	5 ( 9.09%)	6 (3.5%)	0.13
Total septic complications	9 ( 16.4%)	13 (7.6%)	0.06
Total complications	30 ( 54.5%)	63 (36.8%)	0,06

The study population had a median age of 63 years, with a slight predominance of female patients. Most patients were from urban areas, and tumors were mostly located in the middle rectum. Three patients received chemoradiotherapy, and common comorbidities included hypertension, diabetes, and obesity. Hospitalization ranged from 8 to 28 days. Postoperative complications included five fistulas and three abscesses, with one death related to a fistula. These findings highlight that fistulas were the most frequent serious complication in this cohort, particularly among patients with comorbidities or prior chemoradiotherapy (Table 4).

**Table 4:** Clinical, pathological, and laboratory characteristics of patients undergoing LAR

Baseline patient and fistula/abscess characteristics	n
Median age	63 years old
Sex ratio (male: female)	3:5
Urban/Rural	5:3
Tumor location	1 S, 5 M, 2 I
Chemotherapy/Radiotherapy	3
Comorbidities	HTN/DM/Obesity
Length of hospitalization	8-28 days
Abscess/Fistula	3 abscesses / 5 fistulas
Deaths	1 fistula death

IR, inferior rectum; MR, medium rectum; SR/JR, superior rectum/rectosigmoidian junction; DM, diabities miellitus; obesity; HTN- hypertention

### Summary

Overall, complications were more frequent in the APR group. Early general surgical complications occurred in 7.2% of APR and 12.9% of LAR patients, while early septic complications were 8.0 % vs. 4.1%, respectively. Tardive general complications were similar between groups (14.5% vs 16.4%), whereas tardive septic complications were higher in APR (9.1% vs 3.5%). Total septic complications (16.4% vs 7.6%) and total complications (54.5% vs 36.8%) showed a trend toward higher rates in APR, though differences did not reach statistical significance (P ≈ 0.06).

## Discussion

This retrospective cohort study of 226 patients did not demonstrate a statistically significant difference in septic comorbidities between patients undergoing APR or LAR for rectal cancer. Additionally, there were no statistically significant differences in length of hospital stay or in rates of general postoperative complications. Multiple studies also reported no significant differences between the two groups [20-22]. Nevertheless, sphincter-preserving surgery can be performed on the majority of patients, but the high prevalence of low anterior resection syndrome and its associated chronic sequelae remains clinically relevant [23].

### Surgical outcomes and risk factors

The “gold standard” in rectal cancer surgery is considered anterior resection with mesorectal excision and sphincter preservation. Sepsis was first defined in 1991 at a consensus conference, where infection was recognized as a trigger that could lead to systemic inflammatory response syndrome (SIRS). When this response is associated with organ dysfunction, the condition is classified as severe sepsis, with a high potential to progress to septic shock [13,19].

Our results highlight the seriousness of septic complications following low anterior resection and abdominoperineal resection. Several factors may explain the increased risk of AL. The most frequently analyzed variables associated with this risk include hypertension, sex, BMI, and tumor location. The American Society of Anesthesiologists (ASA) score also identifies predictive factors, including significant weight loss, number of stapler firings, surgical approach, intraoperative blood loss, transfusions, type of surgery, operative time, and the interval between neoadjuvant chemotherapy and surgery [24]. AL is associated with reintervention, prolonged hospital stay, increased risk of permanent stoma, higher recurrence rates, and poorer overall outcomes [25]. A multivariate meta-analysis of 20 retrospective studies including 4,764 patients (61.6% male; median age 63 years) demonstrated that AL is associated with local inflammation, leading to fibrosis and bowel narrowing, which adversely affects bowel motility [26]. Although tumor stenosis has been reported in the literature as a predictor of recurrence and AL, this association was not statistically significant in our study [27].

AL was detected in 14% cases, increasing the risk of low anterior resection syndrome, which may lead to incontinence, urgency, tenesmus, and incomplete bowel evacuation [22]. In the present analysis, AL in the LAR group occurred in 4.6% of patients and was classified as early (<7 days) or tardive (7–30 days), with one death resulting from early septic complications.

Hypertension, diabetes mellitus, and obesity were identified as predictive factors for AL in this study. The vascular network is located in the third layer of the rectal wall, corresponding to T3 parietal infiltration according to the TNM classification [28]. Hypertension increases the risk of AL by promoting microvascular disease and ischemia, thereby impairing anastomotic healing [29]. Adequate blood pressure control before surgery is therefore essential. Elderly patients with cardiovascular disease are also more likely to develop AL compared with those without cardiovascular disease [13].

Diabetes mellitus significantly increases the risk of AL in patients undergoing rectal cancer surgery. The risk is likely due to chronic macrovascular and microvascular changes, as well as acute stress-induced hyperglycemia during surgery. A meta-analysis including 5 studies with a total of 93,173 diabetic patients reported significantly higher rates of risk of surgical site infection, urinary complications, AL, and postoperative complications [29].

Obesity is another significant risk factor for AL, particularly in LAR. Elevated insulin levels and pro-inflammatory mediators may impair anastomotic healing, and technical challenges in obese patients further increase surgical complexity [30,31].

### Role of multimodal treatment and patient optimization

Sarcopenia is associated with higher chemotherapy toxicity and poorer outcomes. Patients with higher lean body mass (LBM) demonstrate better tolerance and improved results. Chemotherapy regimens may promote skeletal muscle depletion through pro-atrophic mechanisms, mitochondrial dysfunction, and reduced protein synthesis. Pro-anabolic, muscle-targeted strategies have shown promise in preserving muscle mass in cancer patients [32].

Indocyanine green (ICG) fluorescence imaging is a rapid and cost-effective technique for assessing bowel perfusion intraoperatively. It provides real-time visualization of rectal wall vascularization at the anastomotic site and has been associated with reduced rates of severe complications such as AL, pelvic abscess, and delayed initiation of adjuvant therapy [17].

Prehabilitation is a new approach to improve patients’ physical and nutritional status, as well as their psychological well-being, before surgery. This comprehensive review summarized the currently available data on prehabilitation in the management of colorectal cancer [33]. Even though the majority of studies were not homogeneous in their design and interventions, most showed at least some benefits: improved physical performance and nutritional status, reduced length of hospital stay and postoperative complication rate, and improved quality of life. However, more research on optimal prehabilitation techniques is needed to establish the best prehabilitation strategy for managing colorectal cancer patients [34]. Even though prehabilitation is a new and promising approach to optimize patients before surgery, total mesorectal excision with rectal preservation remains the gold standard treatment for rectal cancer. Studies show that close distal resection margins are oncologically safe after neoadjuvant chemoradiotherapy. The risk of anastomotic leakage continues to be strongly influenced by patient comorbidities; a diverting stoma should be considered in patients with obesity [35,36].

The aim of radiotherapy in rectal cancer is to enable curative treatment in more advanced cases or to reduce the risk of recurrence in early-stage disease (neoadjuvant therapy), nevertheless the oncological outcomes may be affected also by the specific resection approach used during total mesorectal excision (TME) for rectal cancer. Radiotherapy in rectal cancer aims to improve resectability in advanced disease and reduce recurrence risk in early-stage tumors. In the present study, no statistically significant differences were observed among radiotherapy doses (25 Gy, 45 Gy, 50.4 Gy), although tumor regression was noted in all groups. Similar findings have been reported in comparable prognosis between 45 Gy versus 50.4 Gy regimens, notably an increased risk of gynecological and decreased risk of prostate cancer was found in the radiotherapy group [37-42].

### Comparison of APR and LAR mortality and morbidity

Through this analysis, we conclude that although patients undergoing APR may experience complications such as parastomal hernia and delayed perineal wound healing, those treated with LAR may face issues including incontinence, anastomotic leakage, and low anterior resection syndrome. Additionally, APR requires lifelong stoma care. From a cost-effectiveness perspective, LAR offers advantages when oncologically feasible, as it preserves sphincter function and maintains bowel continuity. Adjusted odds ratio analysis demonstrated no significant differences in septic complications between the two groups. However, the overall number of complications was slightly higher in the LAR group, including one death due to anastomotic leakage. Despite this, meta-analyses of oncological outcomes have reported more favorable long-term results in patients undergoing LAR compared with APR [20,40,43,44].

### Limitations

The present study has several limitations. First, it was conducted in a non-emergency center, and we were unable to review the records of patients who may have presented with fistula or other major complications at specialized emergency hospitals. As a result, some postoperative septic events may not have been captured.

Second, there was non-uniformity in surgical approach, including both open (conventional) and laparoscopic procedures. Additionally, heterogeneity among surgeons may have influenced treatment decisions. Some surgeons preferred ultra-low anastomoses, whereas others opted for abdominoperineal resection for tumors located in similar anatomical positions, potentially introducing selection bias.

Furthermore, Hartmann’s procedures and anterior resection were included to account for potential anastomotic leakage, whether the anastomosis was performed during the initial surgery (LAR/AR) or after restoration of bowel continuity (reversal of Hartmann’s procedure). This inclusion may have introduced additional variability in outcome assessment.

## Conclusion

Rectal surgery remains associated with a considerable incidence of septic complications. The risk of fistula is higher in the distal rectum (anastomosis below the Douglas pouch), a region also characterized by a higher occurrence of pelvic abscesses. Moreover, complications arising in the distal rectum tend to be more severe and are associated with greater morbidity and mortality compared with those occurring in the upper rectum or rectosigmoid junction. Data from this study indicate that the incidence of loco-regional septic complications is strongly associated with patient comorbidities and surgical technique and approach. No statistically significant difference in septic complications was observed between patients undergoing APR and those undergoing LAR. Identification of prognostic factors for septic pelvic complications following anterior rectal resection may allow the development of a predictive risk score. Such a tool could help identify patients at high risk for fistula or abscess, enabling more individualized surgical strategies and potentially improving postoperative outcomes. Overall, these findings support the use of low anterior resection whenever oncologically feasible in the curative treatment of rectal cancer.
